# Polyvinyl alcohol nanoparticles loaded with propolis extract: Fabrication, characterization and antimicrobial activity

**DOI:** 10.5599/admet.1740

**Published:** 2023-05-18

**Authors:** Benazir Subaşı-Zarbaliyev, Gozde Kutlu, Fatih Tornuk

**Affiliations:** Yildiz Technical University, Faculty of Chemical and Metallurgical Engineering, Department of Food Engineering, Davutpasa Campus, Istanbul, Turkey

**Keywords:** Encapsulation, electrospraying, thermal property, antimicrobial activity, morphological properties

## Abstract

**Background and Purpose:**

Propolis has high potential beneficial bioactive properties such as anti-oxidative, antimicrobial, and anti-tumour activities. However, the bitter taste and the insolubility nature of propolis in water lead to some limitations in their usage in functional food applications.

**Experimental Approach:**

Herein, we evaluated the effects of nanoencapsulation of propolis at the different concentration levels (0, 0.4, 0.8, 1.0, and 1.2 %) into the polyvinyl alcohol (PVA) nanoparticles using the electrospraying method, on the structural, physical, antioxidant, antimicrobial and thermal properties.

**Key Results:**

The results revealed that the fabricated nanocapsules (PVA-NPs) obtained under optimal conditions had uniform size distribution and unstable particles with small particle size between 104-258 nm, a polydispersity index <0.317, and a zeta potential between -5 and +5 mV. The maximum encapsulation efficiency of PVA-NPs was about 25.32 % for 1 % of the initial propolis loading level. DSC thermal experiments showed an increase in the thermal stability of the propolis loaded PVA nanoparticles as compared to the neat PVA nanoparticles. The percent inhibition of DPPH radical scavenging activity of the nanocapsules was between 80 and 89 %. SEM analysis revealed that PVA-NPs had a spherical shape with a rough surface and were composed of long and thin fibres at nanometric diameters. FT-IR analysis showed that no indications of any chemical reactions were found between the constituents of the core and wall material due to their physical mixing. Antibacterial efficacy was evaluated by the Broth dilution method and PVA-NPs exhibited good inhibitory activity against *S. aureus* at low concentration ratios, whereas it had no inhibitory activity against *E. coli* O157:H7.

**Conclusion:**

PVA-NPs fabricated using the electrospraying technique can be used for the development of a new promising natural and bioactive agent in the food and pharmaceutical industry.

## Introduction

Materials with reduced size up to nanoscale can display quite different properties as compared to micro or macro-scale equivalents. Therefore, nanotechnology has become one of the most efficient and popular topics with a wide range of application fields such as medical, agricultural and food science. One of the most common research and development areas in nanotechnology applications in the food and pharmaceutical industry is the encapsulation of bioactive compounds. Encapsulation can be defined as a process that is the entrapment or coating of a pure material or a mixture (core material, actives, fill, internal phase, or payload) into a coating material (the capsule, wall material, membrane, carrier or shell) [1,2]. The electrospraying (electro-hydrodynamic atomization), a nanoencapsulation technique directed by the electrostatic forces leading to liquid atomization, has found application on laboratory and industrial scales for the fabrication of the nanoparticles [3]. The main factors affecting the electrospraying process include solution (*e.g.*, viscosity, conductivity, molecular weight, and surface tension), process (*e.g.*, applied electric field, tip to collector distance and feeding or flow rate), and environmental conditions (*e.g.*, the humidity and temperature of the surroundings) [4]. This technique can produce stable nanocapsules with enhanced bioactive properties. Moreover, this technique is simple, promising, one-step, highly reproducible and usable for bulk applications. It is also possible to set process parameters and produce more uniform particles with lower particle sizes and higher encapsulation efficiency [5].

Polyvinyl alcohol (PVA) is a water-soluble, odourless, tasteless, non-toxic, non-carcinogenic and fully biodegradable synthetic polymer obtained by partial or complete hydrolysis of polyvinyl acetate. It forms an excellent particle and fibre structure either alone or when blended with other natural polymers and offers a great promise for better biocompatibility, particularly due to its good chemical resistance, stability and flexibility. Also, it shows stability in a wide range of temperature and pH [6,7].

Propolis (bee glue) is a natural honeybee product with a naturally heterogeneous complex matrix used for the construction, maintenance, insulation, and protection of hives by the honeybees from various plant sources [8]. It is a sticky, balsamic and resinous material that can vary in colour from light yellow to dark brown and has a sweet but also bitter taste [9]. Its composition can vary depending on its botanical and geographical origin [8]. Additionally, it has a great potential for health benefits in terms of its antiseptic, antibacterial (particularly against Gram-positive bacteria), anti-mycotic, anti-viral, anti-protozoal, anti-oxidative, anti-inflammatory, and anti-tumour properties [10]. Although it has unique beneficial health effects, its use in its original form is limited due to its low solubility in water and organic solvents and its strong flavour [11]. To date, several fabrication techniques such as spray-drying [12,13], electrospinning [11], complex-coacervation [14], electrospraying [15], and pressurized carbon dioxide anti-solvent crystallization [16] have been used for propolis encapsulation to overcome solubility problems. To the best of our knowledge, no published papers have focused on the encapsulation of propolis into the PVA by the electrospraying method. Therefore, the purpose of the current work was (i) to optimize the nanoencapsulation conditions of propolis using PVA at the highest encapsulation efficiency and to make their characterization by their antioxidant (DPPH scavenging), antimicrobial, morphological (SEM), thermal (DSC), and structural (FT-IR) properties.

## Experimental

### Materials and reagents

Raw propolis used in this study was obtained from Arifoğlu Spice& Food Industry Trade Company (İstanbul, Turkey). Polyvinyl alcohol (Sigma-Aldrich Company, Germany), nutrient broth (Merck, Germany), Maximum Recovery Diluent (MRD, Merck, Germany) and 2,2-Diphenyl-1-picrylhydracyl (DPPH) (Sigma-Aldrich Company, Germany) were purchased. Methanol and ethanol were analytical grades.

### Preparation of propolis extract

Propolis extract was obtained by using 80 % ethanol as solvent. Firstly, 8 % of ethanolic propolis solution was prepared. The obtained solution was stirred with a magnetic stirrer (IKA C-MAG HS 7, Germany) for 24 h at room temperature and then kept at -20 °C for 3 days to precipitate the impurities. After that, the supernatant was taken and filtered through filter paper. Following the filtration, the solution was first centrifuged (Centrifuge Multifuge X3 FR, Thermo Scientific) twice at -5 °C for 5 min at 4.200 rpm. Afterwards, the supernatant was filtered by passing through a 0.22-μm filter. Removal of the solvent from ethanol extract was done by vacuum evaporation (Heidolph Rotary Evaporator, Laborota 4000, Schwabach, Germany) at 50°C. The resulting propolis extract was kept in the refrigerator at 4 °C until further use.

### Optimization of electrospraying conditions based on PVA concentration

In this study, prior to propolis encapsulation, optimum PVA concentration was determined to obtain homogeneous and low-diameter PVA nanoparticles using the electrospraying technique. For this purpose, different PVA solutions were prepared at various concentrations to determine the most appropriate PVA concentration and to optimize the electrospraying conditions and then the nanoparticles were fabricated depending on the different processing conditions using the electrospraying method. In light of the obtained results of the size and zeta potential measurements, optimization studies were performed.

### Preparation of the coating material

Different aqueous PVA solutions 5 to 10 % (w:v) were prepared and each solution was shaken in a water bath (Memmert Model SV 1422, Schwabach, Germany) at 100 rpm for 20 min at 50 °C to form a homogeneous solution. The nanoparticles were fabricated using an electrospraying device (Nanodev Company, Ankara, Turkey). For this purpose, 5 mL plastic syringes (Steriject, with a 0.80038 mm diameter needle), each filled with neat PVA feed solutions, were inserted into the syringe pump (New EraPump Systems Inc., NE-300, Hauppauge, NY) to spray the feed solutions. During electrospraying, different process parameters, such as voltage (10-20 kV), solution flow rate (0.3-0.4-0.5 mL h^-1^), and the tip-to-collector plate distance (10-20 cm), were tested. The most suitable processing parameters were determined by measuring the particle size and assessing the microscope images of resulting nanoparticles applied to the different process parameters mentioned above.

### Particle size measurements of neat PVA nanoparticles

A Malvern Zetasizer Nano ZS instrument (Malvern Instruments Co. Ltd., Worcestershire, UK) was used to determine the variations in the particle size and polydispersity index (PDI) values depending on the PVA concentration in the feed solutions (5, 6, and 10 %; w/v). For this purpose, the nanoparticles were diluted with sterile distilled water, homogenized using an Ultra-Turrax (Daihan, HG-15D, Gang-Won-Do, South Korea) for 30 min, and then passed through 0.45 μm pore size disposable filter. Measurements were carried out in triplicate.

### Fabrication and characterization of PVA nanoparticles loaded with propolis extract (PVA-NPs)

A slightly modified procedure reported by Durán *et al.* (2007) [17] was followed to fabricate PVA-NPs with optimization of the fabrication parameters outlined below. Firstly, PVA solutions containing propolis extracts at different concentrations (0, 0.4, 0.8, 1.0, and 1.2 %) were prepared. For this purpose, 10 mL of 6 % PVA solution was prepared and placed in a water bath (Memmert Model SV 1422, Schwabach, Germany) with constant shaking at 80 °C for 60 min. Then, the propolis extract was added and stirred for 24 h at ambient temperature with a magnetic stirrer (IKA C-MAG HS 7, Germany) to obtain a completely homogeneous solution. Afterwards, the solutions were filled into the 5 mL plastic syringe fitted with a steel needle (Steriject, with a 0.80038 mm (diameter(length) needle) and fed into the electrospraying device applying the following process parameters: voltage, 15 kV; the tip to collector plate distance, 10 cm; and solution flow rate, 0.5 ml/h. Eventually, VA-NPs were collected from the aluminium foil-coated plate. The samples were coded as PVA-NP0, PVA-NP1, PVA-NP2, PVA-NP3 and PVA-NP4 based on propolis concentrations of 0, 0.4, 0.8, 1.0, and 1.2 %, respectively.

### Surface charge (ζ-potential) and particle size measurements

Particle size and zeta potential measurements of PVA-NPs were measured using a Malvern Zetasizer Nano ZS analyser (Malvern Instruments Co. Ltd., Worcestershire, UK) at 25 °C. For this purpose, PVA-NPs were diluted with distilled water and the measurements were conducted. Each result was calculated as the average of 10 (one set of 10) measurements. The results of particle size and *ζ*-potential were presented as nanometre and millivolt, respectively.

### Determination of encapsulation efficiency

The encapsulation efficiency of PVA-NPs was determined according to a method described by Durán *et al.* [17], with some modifications. PVA-NPs containing 10 ml of deionized water were continuously stirred for 60 min using a magnetic stirrer and centrifuged (Centrifuge Multifuge X3 FR, ThermoScientific) twice for 5 min at 5.000 rpm. Then each supernatant was filtered through the filter papers, the absorbance measurements were done using a UV visible spectrometer (UV-1800, Shimadzu, Kyoto, Japan). The percent encapsulation efficiency was determined using the following equation.


(1)

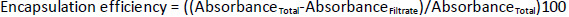



### FT-IR

Molecular characterization of the propolis extract and PVA-NPs were performed using a Bruker FT-IR spectrometer model Tensor 27 (Bremen, Germany) coupled to a diamond platinum ATR unit (spectral range of 400-4000 cm^-1^, resolution: 4 cm^-1^, and scans: 32).

### DSC

Thermal properties of PVA-NPs were determined using a DSC instrument (DSC, Q100, TA Instruments Inc., New Castle, DE, USA) in the temperature range of 40-400 °C at a heating rate of 10 °C min^-1^ under a nitrogen atmosphere. About 4-5 mg of PVA-NPs sample was placed on aluminium-coated pans and loaded into the instrument chamber at ambient temperature. An empty pan was used as a reference.

### DPPH radical scavenging activity

Firstly, 1.5 mL of ethanolic extract of PVA-NPs was well-mixed with 1.5 mL of DPPH solution, vigorously vortexed for 1 min and left in a dark environment for 30 min at room temperature. Then the absorbance values were measured at 517 nm using a UV-visible spectrometer (UV-1800, Shimadzu, Kyoto, Japan). The DPPH scavenging activity was calculated using the following equation:


(2)

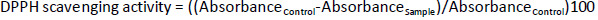



### Antimicrobial activity

The Broth dilution method described by Feyzioglu *et al.* [18] was used for the determination of the antimicrobial activity of PVA-NPs against two different bacterial strains (*S. aureus (*ATCC 25923) and *Escherichia coli* O157:H7 (ATCC 33150)). Initially, after activating bacterial strains twice in nutrient broth at 37 °C for 24 h, the test tubes, including 7 mL of nutrient broth, were seeded with the bacterial strains (a final inoculum of approximately 10^8^ colony-forming units (cfu mL-^1^) and then incorporated with2 % of the PVA-NPs samples. Following incubation of the test tubes for 5 h in the shaking incubator at 37 °C at 140 rpm, serial dilutions were prepared, and colony counts were analysed using a spread plating method. Colony forming units were counted after 24 h of incubation of Petri plates at 37 °C. The results were presented as logarithmic values.

### SEM

The surface morphology of PVA-NPs was analysed by scanning electron microscopy (SEM, FEI Quanta FEG 250, USA) under vacuum at 20 kV and at a 7.6 mm of working distance. Prior to analysis, the samples were lyophilized and coated with gold.

### Statistical analysis

A minimum of three independent experiments with two replicates were performed to achieve efficient results and the findings were tabulated as mean ± standard deviation. A one-way ANOVA (analysis of variance) test for the determination of the statistical differences between the data was applied using a *t*-test at the *p*<0.05 level and the statistical analysis of data was performed by the JMP software (v6; SAS Institute Inc., Cary, NC, USA).

## Results and discussion

### Optimization of electrospraying processing conditions

In this study, firstly, different concentrations (5, 6 and 10 %) of PVA solutions were fed to the electrospraying system. The particle size of the nanoparticles was monitored. The highest particle size value was measured in the solution containing 10 % PVA (212-255 nm), followed by the solution containing 5 % PVA (144-180 nm), and the solution including 6 % PVA (86-106 nm). Microscope images showed the formation of nanofibers at higher concentrations than 7 %, while dark (black) beads were obtained at lower concentrations (5 %). Moreover, laboratory trials showed that an increase in the ultrasonication time before the electrospraying led to the formation of more homogeneous particles. Overall, ultrasonication frequency and coating solution concentration were determined as 50 Hz and 6 %, respectively, in this study. Moreover, optimum electrospraying conditions were an applied voltage of 15 kV, a tip-to-collector plate distance of 10 cm and a solution flow rate of 0.5 ml / h.

### Characterization of PVA-NPs

Following the optimization of electrospraying conditions, PVA-NPs were prepared with the incorporation of propolis extract. Then the resulting nanoparticles were characterized.

### ζ-Potential, PDI and particle size

The summary of the ζ-potential, PDI and mean particle size findings for the PVA-NP0, PVA-NP1, PVA-NP2, PVA-NP3 and PVA-NP were given in Table 1. The different wall materials and types of encapsulating agents influence the various capsule characteristics (*e.g.*, particle size, encapsulation efficiency, oxidative stability, and morphology) [19].

PVA-NP0 had lower average particle size values (106.37 nm) compared to the other formulations. The average particle size of PVA-NPs loaded with the aforementioned five concentrations of propolis was found to be between 106.37 and 258.51 nm. Moreover, an increase in the loaded propolis ratio led to an increase in the average particle size except for PVA-NP4. However, no significant differences were found between PVA-NP1, PVA-NP2 and PVA-NP4 (p>0.05).

The ζ-potential is a very good index for the specification of the surface electrical status (the electrostatic or charge repulsion/attraction) of colloidal systems. This parameter was used for the evaluation of the encapsulation of the core material in the nanoparticle and the stability or aggregation of the nanoparticle in the suspension. All the *ζ*-potential values were in the -0.11 to 3.09 mV range with no statistically significant influence of propolis ratio between PVA-NP0/PVA-NP1 or PVA-NP2/PVA-NP3/PVA-NP4 (*p*>0.05). In the current study, PVA-NPs had zeta potential values near zero with slight differences depending on the propolis concentration. Increasing propolis caused a more negative surface potential, while the highest ζ-potential was measured in PVA-NP0 (3.09 mV). The *ζ*-potential values between -30 and +30 mV are considered as the formation of stable water suspensions [13], showing that the fabricated PVA-NPs formed unstable colloidal dispersions due to the low electrostatic repulsion. *ζ*-potential was lower (more negative) as the propolis ratio increased, but the variations were not very high. Similar findings were reported by Baysan *et al.* [20], who found the *ζ*-potential values of the propolis-loaded capsules prepared with different coating material combinations (whey protein isolate, gelatin, Na-caseinate, maltodextrin, lactose, and gum arabic) in the range of -6.16 to 0.42 mV. The reduction in diameter from PVA-NP4 to PVA-NP0 was probably due to the changing surface charge of the PVA-NPs and the agglomeration of nanoparticles in an aqueous medium.

PDI is one of the best indicators to evaluate the particle size distribution in suspensions and estimate the physical and rheological features of the colloidal systems (*e.g*., stability, the release of core material and encapsulation efficiency) [21]. Lower PDI means more homogeneous particle size distribution. PDI values of the samples varied from 0.22 to 0.48 (Table 1), indicating that the fabricated nanoparticles were relatively homogeneous samples with intermediate PDI. We can interpret the particle size distribution of PVA-NPs as extremely polydisperse nor broad, as previously noted by Wrona *et al.* [22]. The use of different concentrations of propolis had a significant effect on PDI (*p*<0.05), but PVA-NP3 and PVA-NP4 exhibited statistically no significant differences (*p*>0.05). Additionally, PVA-NP0 and PVA-NP2 had a more homogenous particle size distribution as compared to the other PVA-NPs. In a previous study, particle size (200-280 nm) on a nanometric scale, the *ζ*-potential values (-12.7 to -33.5 mV) and PDI (0.089 to 0.169) for the polymeric nanoparticles loaded with Brazilian red propolis extract were determined by Do Nascimento *et al.* [23]. One recent research also reported the average particle (208.1-362.2 nm), PDI (0.23-0.26) and *ζ*-potential (-26.9 to -31.7 mV) of propolis-loaded zein/caseinate/alginate nanoparticles [24]. Our results showed that the fabricated PVA-NPs could be used for food and pharmaceutical purposes due to their small average particle size and moderate PDI values (<0.4), as also supported by Gonçalves and co-workers [25].

### Encapsulation efficiency

The encapsulation efficiency gives information corresponding to the retention degree of the core material within the hollow structure of the coating material(s) compared to the reference core amount incorporated into the encapsulation system. PVA-NP3 and PVA-NP1 had the highest (25.32 %) (*p*<0.05) and the lowest (5.09 %) encapsulation efficiency values, respectively (Table 1). Increasing propolis concentration resulted in higher encapsulation efficiency values up to 1 %, then decreased. This indicates that the excessive use of core material does not always give a better encapsulation yield [26]. Similar findings were reported by Durán *et al.* [17], who found encapsulation efficiency levels around 25 % for poly(e-caprolactone) microparticles loaded with propolis extract. In another study conducted by Baysan *et al.* [27], the encapsulation efficiency values of maltodextrin and whey protein isolate powder particles containing propolis extract were between 29.79 and 99.73%. Additionally, Zhang *et al.* [24] used zein/dissociated sodium caseinate micelles/alginate nanoparticles for the encapsulation of propolis and noted the encapsulation efficiency as 76.2-86.5 %, depending on the applied method. Ina recent study conducted by Shakoury *et al.* [28], the encapsulation efficiency of whey protein nanoparticles loaded with propolis extract was in the range of 65.49 to 84.03 %.

### Structural characterization by ATR-FT-IR

**Figure 1.**
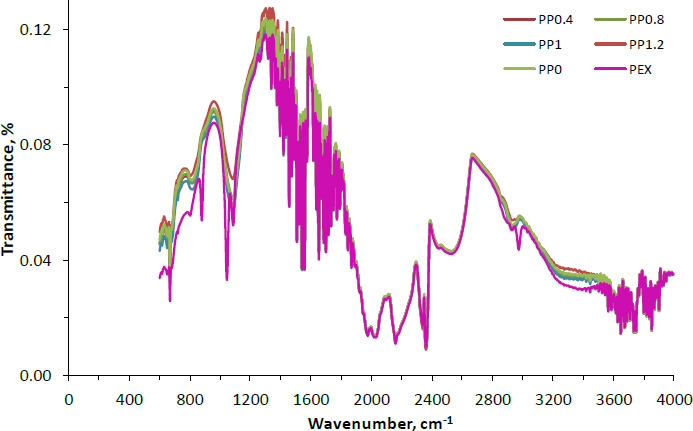
FTIR spectra of the PVA-NPs; PVA: polyvinyl alcohol, PEX: propolis extract, PVA-N0: neat PVA nanoparticles, PVA-N1: PVA nanoparticles loaded with propolis in the ratio of 0.4 %, PVA-N2: PVA nanoparticles loaded with propolis in the ratio of 0.8%, and PVA-N3: PVA nanoparticles loaded with propolis in the ratio of 1.0 %, and PVA-N4: PVA nanoparticles loaded with propolis in the ratio of 1.2 %.

The FTIR spectra of PVA-NP0, PVA-NP1, PVA-NP2, PVA-NP3 and PVA-NP4 obtained in the range 4000 to 400 cm^-1^ were given in Figure 1. The wavelengths near 3336 and 1347 cm^-1^ could be related to the phenolic hydroxyl groups [29].

The wavelengths found at 1725-1705 cm^-1^ can be ascribed to the C=O stretch in aliphatic ketones [23]. The band at 1670 to 1600 cm^-1^ could be related to the C=C stretch of conjugated aromatic rings [30]. The observed bands near 1400 and 1340 cm^-1^ correspond to CH_2_ bending, OH in-plane bending, and the C-H deformation (CH_3_ or O-H in-plane bending) [31]. Additionally, the weak peak of the stretch of aromatic ether C-O bond (for flavonoids) appeared around 1045 cm^-1^. The propolis extract spectra also exhibited the band’s disappearance at 1043 cm^-1^ from PVA-NPs. Furthermore, corresponding to the PVA-NPs, no new bands were determined. In the FTIR spectra of the propolis extract, the wavelength around 1045 cm^-1^ shifted to the higher band (1083 cm^-1^) with the fabrication of PVA-NPs. Moreover, molecules containing the plane of aromatic C-H regarding the angular deformation were attributed to near 877 cm^-1^ [32]. Similar FT-IR spectra were also given for the polymeric nanoparticles loaded with Brazilian red propolis extract by Do Nascimento *et al.* [23]. Overall, it can be inferred from the FTIR spectra that the encapsulation was successfully performed and a good interaction between the constituents of the system was achieved.

### Thermal characterization by DSC

**Figure 2.**
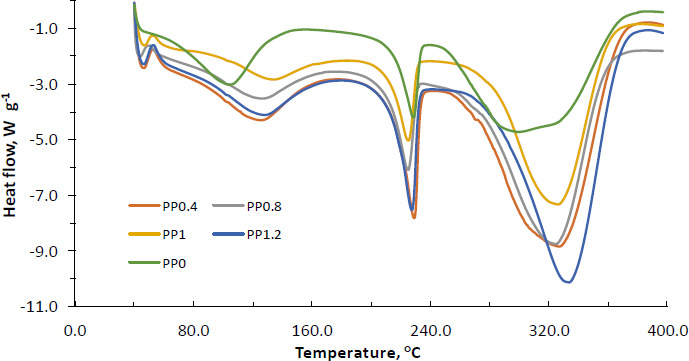
DSC thermograms of the PVA-NPs; PVA: polyvinyl alcohol, PVA-N0: neat PVA nanoparticles, PVA-N1: PVA nanoparticles loaded with propolis in the ratio of 0.4 %, PVA-N2: PVA nanoparticles loaded with propolis in the ratio of 0.8 %, and PVA-N3: PVA nanoparticles loaded with propolis in the ratio of 1.0 %, and PVA-N4: PVA nanoparticles loaded with propolis in the ratio of 1.2 %.

The thermographs of PVA-NP0, PVA-NP1, PVA-NP2, PVA-NP3, and PVA-NP4 were illustrated in Figure 2 and glass transition temperature (*T*_g_), onset temperature (*T*_o_), peak temperature (*T*_p_) and ending temperature (*T*_e_) values were determined. As can be seen, while the DSC thermographs of PVA-NP1, PVA-NP2, PVA-NP3, and PVA-NP4 were similar, whereas PVA-NP0 possessed a broader endothermic peak from 242 °C to 390 °C with a maximum temperature of 305 °C. It can be due to the changes in thermal characteristics of PVA with the addition of propolis. The PVA-NP0 depicted the three endotherm events with *T*_o_ at 44, 150 and 242 °C, *T*_p_ at 105, 225 and 305 °C and *T*_e_ at 150, 237.5 and 390 °C and its corresponding *T*_g_ was determined at 50 °C.

Encapsulation of propolis extract into the PVA polymer matrix resulted in four endothermic peaks. Our findings supported the results obtained by do Nascimento *et al.* [23], who also observed four endotherm events when Brazilian red propolis extract was encapsulated into the polymeric nanoparticles. Additionally, the formation of extra small endothermic peaks at around 40-45 °C could be due to the little crystalline nature of the particles. It was also determined that *T*_o_ of the third endothermic peak for PVA-NP0 shifted to higher temperatures after encapsulation of propolis, which implied that encapsulation supplied thermal protection of propolis. Variations of peak positions indicated the interactions between PVA and propolis extract that led to the formation of new structural organizations. Among the tested PVA-NPs, in general, PVA-NP1 and PVA-NP4 exhibited similar endothermic peaks with lower temperature values, while PVA-NP3 showed endothermic peaks at greater degrees (*T*_p_ of 45, 137, 223, and 325 °C). Moreover, the DSC thermogram of the PVA-NP2 illustrated four endothermic peaks at *T*_p_ of 40, 128, 223, and 329 °C. Nascimento *et al.* [23] reported that the observed endothermic peak around 135 °C for the polymeric nanoparticles loaded with Brazilian red propolis extract was related to the fusion processes of low molecular weight compounds such as flavonoids in the mixture and other phenolic compounds found in the propolis extract. The findings revealed that propolis-loaded nanoparticles were decomposed at a higher temperature as compared to their neat forms, showing that the thermal stability of PVA-NPs was enhanced by the incorporation of propolis into the PVA polymer matrix.

### DPPH radical scavenging activity

The DPPH scavenging activity (Table 1) of the PVA-NPs was found in the following order: PVA-NP4 (89.25 %) > PVA-NP3 (87.32 %) > PVA-NP2 (85.09 %) > PVA-NP1 (78.13 %) >PVA-NP0 (0 %), showing that the DPPH-radical scavenging abilities of the samples were correlated with the loaded propolis level. The findings showed that the neat PVA nanoparticle formulation (PVA-NP0) possessed no antioxidant activity. Additionally, no significant differences were determined in the radical scavenging activities of PVA-NP3 and PVA-NP4 (*p*>0.05). Our findings were supported by the results obtained by Aytekin *et al.* [33], who reported that DPPH scavenging activities of various propolis liposomal formulations varied from 76.21 to 100.3 %. In another study conducted by Andrade and co-workers [34], DPPH inhibition levels of the microcapsules containing spray-dried Brazilian brown, green and red propolis were found between 82.00 and 89.00 %.

### Antibacterial activity

The antibacterial activity of PVA-NP0, PVA-NP1, PVA-NP2, PVA-NP3 and PVA-NP4 were tested against two pathogenic bacterial strains (*E. coli* O157:H7 and *S. aureus*) and the findings were given in Table 2. The type of PVA-NPs and the bacterium strain affected the bacterium growth inhibition. PVA-NP0, which does not contain propolis did not show any inhibitory activity against any of the pathogens, which shows the ineffectiveness of PVA itself. Among the studied pathogenic strains, propolis-loaded PVA-NPs exhibited stronger antibacterial activity against the Gram-positive *S. aureus*, where a complete elimination was achieved by the effect of PVA-NP2, PVA-NP3 and PVA-NP4. A significant reduction in *E. coli* numbers was also provided by the application of propolis-loaded PVA-NPs. In a previous study, Przybyłek and Karpiński [35] reported that the propolis extract had more bacterial inhibition capability on Gram-positive strains as compared to the Gram-negative bacteria. This could be due to variations in the structural composition of cells and the cell wall of Gram-positive and Gram-negative bacteria [33,36].

The results also demonstrated that propolis extract kept its antibacterial activity after nanoencapsulation. The findings regarding *S. aureus* agreed with the observations reported by previous researchers [37,38]. Similarly, Almuhayawi [39] reported that the antibacterial effects of propolis were dependent on several factors, such as the origin of propolis and the type of bacterial strains. The results were also in accordance with those of Lu *et al.* [40], who reported that *S. aureus* was almost susceptible to the ethanolic extract of propolis. The antimicrobial activity of propolis could be due to the presence of hydrophilic and hydrophobic phenolic compounds (*e.g.*, such as flavonoids, aromatic esters and acids) that may directly influence the cell wall of the bacteria [33].

### Morphological characterization by SEM

**Figure 3.**
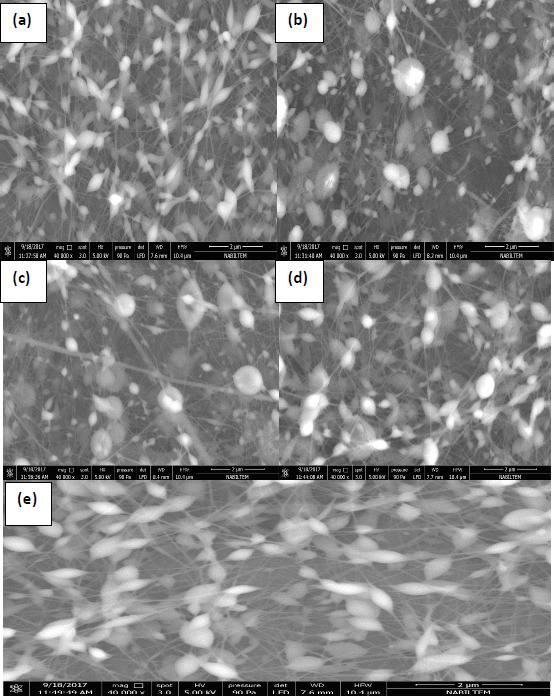
Morphology and size distribution of PVA nanoparticles loaded with propolis in the ratio of 0 % (a), 0.4 % (b), 0.8 % (c), 1.0 % (d) and 1.2 % (e).

SEM images of the PVA-NPs are shown in Figure 3 (a-e). Corresponding to the five different SEM images (magnification 40000e), various particle shapes and mean particle size diameters were seen depending on the five formulations. SEM images indicated no cracks or holes on the surface of PVA-NPs. SEM results clearly revealed that PVA-NPs had a spherical shape with a rough surface and were composed of long and thin fibres at nanometre thickness. The first reason for the formation of nanoparticles and nanofibers in the images could be the applied high voltage to the solutions so-called as Taylor-Cone based spraying effect [41]. According to this theory, the droplets were charged by the high voltage and the surface tension of the polymer solution was overcome by the arising repulsive Coulombic forces. Then, the Taylor cone was formed and when the voltage exceeded the limits, cone-jet formed based on polymer concentration resulting in thin fibre formation [42].

Additionally, another possible reason could be due to the high viscosity of the coating material. Earlier studies reported that the increase in the viscosity of the solution resulted in spindle-like droplets with pure fibres [43]. SEM examination showed that the mean particle size diameter of the PVA-NPs was measured in the range of 100 to 250 nm, confirming the particle size determined by the dynamic light scattering analysis. Although we determined that the dispersions of PVA-NPs were uniform and homogeneous based on the PDI measurement results, SEM photographs showed that the PVA-NPs were non-homogenous. This can be linked with the quickly absorbed moisture from humid air by the PVA-NPs and this phenomenon led to the particle agglomeration.

## Conclusions

Propolis has been popular in recent years for its health-related and pharmaceutical properties. However, it cannot be consumed in its original form due to its bitter and unpleasant taste. Its insolubility nature in water and the easy loss of its antioxidant activity and phenolic compounds are other limitations of propolis in the food industry. In the current work, propolis at different concentrations (0, 0.4, 0.8, 1.0, and 1.2 %) were encapsulated into PVA polymer matrix using the electrospraying method. The dynamic light scattering analysis showed that the nanoparticles had nanoscale particle size with moderate PDI values and formed unstable colloidal dispersions. Encapsulation efficiency of up to 25 % and high DPPH scavenging activity of up to 89.25 % were found for propolis-loaded PVA nanoparticles. FT-IR analysis showed the successful propolis encapsulation and interaction between the constituents. DSC analysis revealed that the thermal stability of propolis-loaded PVA nanoparticles was higher in comparison to their neat nanoparticle forms. Regarding antibacterial activity, the loaded nanoparticles showed stronger antibacterial activity against *S. aureus* than *E. coli.* SEM images demonstrated that the nanoparticles had a spherical shape with a rough surface and were composed of long and thin fibres with nanometre thickness. Overall, the current work confirmed that nanoencapsulation of propolis using electrospraying technique could offer a new and effective approach to overcome its limitations for utilization in the food and pharmaceutical industries.

## Figures and Tables

**Table 1.** Polydispersity index, mean particle size, stability, encapsulation efficiency and DPPH scavenging activity values of PVA-NPs.

Sample name	Polydispersity index	Mean particle size, nm	*ζ*-potential, mV	Encapsulation efficiency, %	DPPH scavenging activity, %
PVA-N0	0.23 ± 0.16^bc^	106.37 ± 14.84^c^	3.09±0.50^a^	-	0.00 ± 0.00^d^
PVA-N1	0.32 ± 0.16^ab^	137.17 ± 61.82^b^	2.77±0.29^a^	5.09 ± 0.37^d^	78.13 ± 0.43^c^
PVA-N2	0.22 ± 0.03^c^	186.75 ± 8.27^b^	-0.24±0.06^b^	19.43 ± 1.16^b^	85.09 ± 2.82^b^
PVA-N3	0.42 ± 0.12^a^	258.51 ± 90.51^a^	-2.44±1.27^b^	25.32 ± 0.32^a^	87.32 ± 0.21^a^
PVA-N4	0.48 ± 0.08^a^	249.33 ± 41.40^b^	-0.11±0.10^b^	18.15 ± 0.26^c^	89.25 ± 1.22^a^

*ζ*-potential: surface charge, PVA: polyvinyl alcohol, PVA-N0: neat PVA nanoparticles, PVA-N1: PVA nanoparticles loaded with propolis in the ratio of 0.4 %, PVA-N2: PVA nanoparticles loaded with propolis in the ratio of 0.8 %, and PVA-N3: PVA nanoparticles loaded with propolis in the ratio of 1.0 %, and PVA-N4: PVA nanoparticles loaded with propolis in the ratio of 1.2 %.

^a-d^: Means followed by the same lowercase letters in the same column showed no statistical difference at the 5 % significance level applying-test between the tested samples.

**Table 2.** The effects of the PVA-NPs on the growth inhibition of E. coli O157:H7 and *S.aureus*.

Name of strain	Log (antibacterial activity / cfu mL^-1^)					
	Sample					
	Control	PVA-N0	PVA-N1	PVA-N2	PVA-N3	PVA-N4
*E. coli*	8.20 ± 0.10^a^	8.52 ± 0.09^a^	8.15 ± 0.17^b^	8.25±0.11^b^	8.03 ± 0.19^c^	8.12 ± 0.09^b^
*S. aureus*	8.24 ± 0.08^a^	8.06 ± 0.07^a^	4.37 ± 0.33^b^	0.00 ± 0.00^c^	0.00 ± 0.00^c^	0.00 ± 0.00^c^

cfu: colony-forming units, PVA: polyvinyl alcohol, PVA-N0: neat PVA nanoparticles, PVA-N1: PVA nanoparticles loaded with propolis in the ratio of 0.4 %, PVA-N2: PVA nanoparticles loaded with propolis in the ratio of 0.8 %, and PVA-N3: PVA nanoparticles loaded with propolis in the ratio of 1.0 %, and PVA-N4: PVA nanoparticles loaded with propolis in the ratio of 1.2 %.

^a-d^: Means followed by the same lowercase letters in the same line indicates that there was no statistical difference at the 5 % significance level applying-test between the tested samples.
